# ScalaBLAST 2.0: rapid and robust BLAST calculations on multiprocessor systems

**DOI:** 10.1093/bioinformatics/btt013

**Published:** 2013-01-29

**Authors:** Christopher S. Oehmen, Douglas J. Baxter

**Affiliations:** Pacific Northwest National Laboratory, Richland, WA 99352, USA

## Abstract

**Motivation:** BLAST remains one of the most widely used tools in computational biology. The rate at which new sequence data is available continues to grow exponentially, driving the emergence of new fields of biological research. At the same time, multicore systems and conventional clusters are more accessible. ScalaBLAST has been designed to run on conventional multiprocessor systems with an eye to extreme parallelism, enabling parallel BLAST calculations using >16 000 processing cores with a portable, robust, fault-resilient design that introduces little to no overhead with respect to serial BLAST.

**Availability:** ScalaBLAST 2.0 source code can be freely downloaded from http://omics.pnl.gov/software/ScalaBLAST.php.

**Contact:** christopher.oehmen@pnl.gov

## 1 INTRODUCTION

Genome and protein sequence analysis using BLAST continues to be among the most used tools for computational bioinformatics. The continued exponential growth in throughput of sequencing platforms has continued to drive the need for ever-expanding capacity for BLAST (Altschul *et al.*, 1990) calculations to support genome annotation, functional predictions and a host of other foundational analysis for sequence data. Parallel BLAST accelerators have been implemented in the past including mpiBLAST (Darling *et al.*, 2003) and ScalaBLAST 1.0 (Oehmen and Nieplocha, 2006). Parallel BLAST drivers accelerate large lists of BLAST calculations using multiprocessor systems. ScalaBLAST 1.0 used a hybrid parallelization scheme in which the sequence list was statically partitioned among processor pairs (process groups). Process groups performed independent BLAST calculations simultaneously, gaining a degree of speedup on the overall calculation in proportion to the number of process groups used in the calculation. The main limitation of ScalaBLAST 1.0 was the use of *static* data partitioning that did not have fault-resilience properties. By contrast, the main limitation of mpiBLAST is the need for pre-formatting datasets to achieve optimized run-time, sometimes requiring repeated attempts on the same dataset to find the right pre-formatting configuration.

We have addressed these limitations in ScalaBLAST 2.0 by (i) re-implementing the task scheduling layer by introduction of a dynamic task management scheme that (ii) does not require pre-formatting. This technique allows processors to obtain work units independently and at run-time based on their availability. This is a *highly tolerant and fault-resilient approach* that ensures that all processors are doing as close as possible to the same amount of work throughout a calculation. In addition, this implementation allows for continued operation even in the presence of processor or other system failures. This is *critical* for all large-scale calculations and is independent of the code being run because the longer the run and the larger the system, the more likely one is to encounter a component failure during a calculation. As the expected run-time increases, the likelihood of successfully completing the calculation before the next failure tends to zero. We demonstrate near-ideal scaling using ScalaBLAST 2.0 calculations to machine capacity on a Linux cluster having >18 000 compute cores even during process failure events. ScalaBLAST 2.0 can be downloaded freely from http://omics.pnl.gov/software/ScalaBLAST.php.

## 2 METHODS

ScalaBLAST 2.0 is implemented using the NCBI BLAST C toolkit distribution version 2.2.13. This is several years old, but it is very stable, and we have found that large-scale sequence analysis centers prefer such stable versions. ScalaBLAST 2.0 supports the five basic BLAST calculation types—blastn, blastp, tblastn, tblastx and blastx and three different output formats (standard pairwise, tabular and tabular with headers). The next major release of ScalaBLAST will include our own implementation of the BLAST algorithm and will not use the NCBI toolkit.

ScalaBLAST 2.0 depends only on message passing interface (MPI) library, which can be downloaded freely. Tasks in ScalaBLAST 2.0 are managed by a dynamic task scheduler. Each query is considered to be an independent task and is processed by a single compute core. Each task contains the query sequence and the whole target database. At the beginning of the run, a single manager process is selected to control which processes receive which tasks for the duration of the computation. Depending on user-configurable parameters (in the sb_params.in file), the manager will have some number of sub-managers. Each sub-manager will in turn have some number of worker nodes. Each collection of sub-manager and worker nodes is referred to as a task group.

At the beginning of each ScalaBLAST job, files are distributed across nodes at the start of a calculation by the manager. Users can set in the sb_params.in file the relationship between processing elements and their underlying file system *independently from the task group configuration*. The task group defines how many workers are associated with each sub-manager. The notion of how to distribute the files is governed by the *disk group*. The disk group is used to map how many compute cores share a common file system. This control is used to support storing output and input on globally mounted or local file systems or combinations of both.

After file distribution is complete, the manager is responsible for tracking which tasks have been assigned and which tasks have been completed. The manager is also responsible for processing the FASTA input files (both query and target database are in FASTA format, eliminating the need for pre-formatting database files) and distributing these processed files.

The task groups can be controlled by the user and can span multiple compute nodes. For instance, a system with eight-core nodes can have a task group size of 24 in which sets of three nodes work together as a single task group, having one sub-manager core and 23 worker cores.

This dynamic scheduling layer ensures that when processes fail or get loaded down with tasks taking a long processing time, other processes continue to do meaningful work. This allows for highly skewed input sets to be processed as much as possible in an even run-time. Dynamic scheduling is implemented by having the manager ‘hand out’ tasks to sub-managers. Workers completing a task do not write their output until they verify from the manager (via the sub-manager) whether the task has already been checked back in. Workers then request a new assignment from the manager. When all the tasks have been assigned, any workers reporting for new work are given a duplicate task that has not yet been completed. In this way, nodes that fail during a calculation are simply ignored. Any tasks assigned to them will be re-assigned to other workers until one of them completes the calculation.

## 3 RESULTS

ScalaBLAST 2.0 was run on a Linux cluster at Pacific Northwest National Laboratory that has 2310 compute nodes, each having eight cores for a total of 18 480 compute elements. For blastp scaling runs, our query dataset contained 203 200 proteins with widely varying size distribution. Our query list had an average protein length of 175.1 ± 138.5 residues, with a minimum length of eight and a maximum length of 4299 residues. This list was compared against a version of the non-redundant database from NCBI dated May 2010 and containing 12 million reference proteins. Each query sequence was compared to the reference database using blastp with default BLOSUM62 scoring matrix and print option 9.

### 3.1 Scalability results

**Fig. 1.**
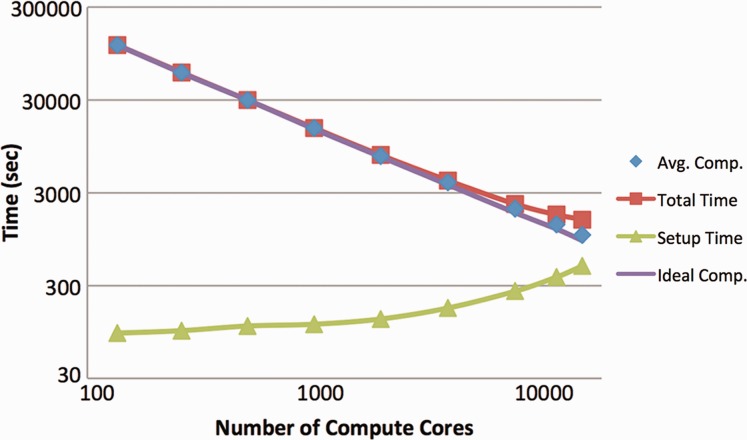
Scaling performance of ScalaBLAST 2.0 on a large protein sequence dataset compared with non-redundant database

Run times include parallel execution startup, time for parsing the input files, creating and distributing their binary counterparts, performing all calculations and terminating the job. Scaling results are shown in Figure 1. This figure demonstrates that for this calculation, ScalaBLAST 2.0 achieved nearly ideal speedup all the way to 16 392 compute cores at which point the whole task list was processed in 27 minutes. We have observed similar scaling performance characteristics for blastn, tblastn, blastx and tblastx program option when using ScalaBLAST 2.0 (results not shown).

### 3.2 Fault resilience

We experienced several examples of *hardware* failure during the course of ScalaBLAST scalability testing. Even in the presence of such failures, ScalaBLAST was able to continue the calculation and complete the task list. We tested the overhead introduced by our fault-resilient design by comparing NCBI BLAST 2.2.13, ScalaBLAST 2.0 running in serial mode and ScalaBLAST 2.0 running in parallel mode with only one worker process. We observed between a 10% improvement to a 24% slowdown in serial processing time when comparing either version of ScalaBLAST 2.0 with serial NCBI BLAST, depending on the dataset and runtime options demonstrating that ScalaBLAST 2.0 scaling is based on the order of magnitude run time for serial execution.

## 4 CONCLUSION

ScalaBLAST 2.0 provides fault-resilient speedup on conventional Linux-based clusters in proportion to the number of nodes in the cluster. On both small- and large-scale systems, this allows users to accelerate the throughput of BLAST calculations that complete even when processes fail in support of robust sequence analysis applications. ScalaBLAST 2.0 can be freely downloaded from http://omics.pnl.gov/software/ScalaBLAST.php.
